# The Occurrence of Seizure Clusters in Patients With Epilepsy Is Partly Determined by Epilepsy Severity: A Single-Center Retrospective Observational Study

**DOI:** 10.3389/fneur.2021.794086

**Published:** 2021-12-09

**Authors:** Rui Zhong, Qingling Chen, Xinyue Zhang, Weihong Lin

**Affiliations:** ^1^Department of Neurology, The First Hospital of Jilin University, Changchun, China; ^2^Department of Hepatology, Second People's Clinical College of Tianjin Medical University, Tianjin, China

**Keywords:** seizure clusters, epilepsy severity, seizure frequency, multiple seizure type, more current ASMs, drug-resistant epilepsy, history of status epilepticus

## Abstract

**Purpose:** This retrospective observational study aimed to investigate the self-reported prevalence of seizure clusters (SCs) in patients with epilepsy (PWE) and its relationship with clinical characteristics.

**Methods:** We retrospectively analyzed data from consecutive PWE from our hospital in northeastern China. Data were collected from the databank of a tertiary epilepsy center. Logistic regression models were employed to investigate the relationships between the individual patient demographic/clinical variables and the occurrence of SC.

**Results:** In total, 606 consecutive PWE were included in the final analysis, and 268 (44.2%) patients experienced at least one seizure cluster. In multivariate logistic regression models, age (OR: 1.014; 95% CI: 1.002–1.027; *p* = 0.02), seizure frequency (OR: 2.08; 95% CI: 1.555–2.783; *p* < 0.001), multiple seizure types (OR: 5.111; 95% CI: 1.737–15.043; *p* = 0.003), number of current anti-seizure medications (ASM) (OR: 1.533; 95% CI: 1.15–2.042; *p* = 0.004), drug-resistant epilepsy (OR: 1.987; 95% CI: 1.159–3.407; *p* = 0.013), and a history of status epilepticus (OR: 1.903; 95% CI: 1.24–2.922; *p* = 0.003) were independent variables associated with a history of SC in PWE.

**Conclusion:** Seizure clusters (SCs) are common occurrences at our study center. The occurrence of SC in individuals with epilepsy, to some extent, is determined by the epilepsy severity.

## Introduction

Epilepsy is one of the most common serious brain disorders, affecting more than 70 million patients worldwide (1). Approximately 30% of patients with epilepsy (PWE) reported uncontrolled or poorly controlled seizures despite having appropriate anti-seizure medication (ASM) therapy (2). Seizure clusters (SCs) refer to a closely grouped series of seizures within a defined period (3). SC is a common clinical phenomenon reported by many PWE (4–7). The reported prevalence of SC ranged from 20 to 83% in prior investigations (6, 8, 9). One major reason for the varying prevalence of SC across the studies is the lack of a precise definition of SC (4, 10). Different clinical definitions of SC have been applied previously, including ≥3 seizures within 24 h, >2 seizures within 24 h, and >2 seizures within 6 h (10).

Many researchers have paid attention to the potential factors contributing to SC in PWE and they also estimated the patients' risk of SC (5, 6, 10). Factors associated with SC in PWE include previous head trauma, extratemporal seizure localization, history of status epilepticus, higher frequency of seizures, intractable epilepsy, symptomatic generalized epilepsy, focal epilepsy (vs. idiopathic generalized epilepsy), epilepsy onset at early age, and longer epilepsy duration (5–7, 10, 11). The use of various SC definitions, different study populations, and the inclusion of PWE with varying degrees of severity of epilepsy may lead to a wide range of reported risk factors across the studies. Furthermore, it has been reported that SC is associated with an increased risk of emergency room visits or hospitalization for PWE (3, 12). The negative effect of SC on the patient's quality of life has also been established (13).

The prevalence of SC in Chinese adults with epilepsy has yet to be investigated, and knowledge about factors associated with SC in PWE is also scarce in mainland China. Thus, our study aimed to answer the following questions: (1) How many Chinese adults with epilepsy have experienced a history of SC defined as ≥3 seizures in 24 h? (2) Which demographic or clinical characteristics are associated with a history of SC? (3) Are the potential risk factors for SC vary by epilepsy type?

## Methods

### Study Design and Study Population

We conducted a retrospective observational study of PWE in an epilepsy management program at the epilepsy center in the First Hospital of Jilin University. A clinical databank was established for PWE in our center. The databank included demographic and clinical information on the PWE who were treated and followed up at our hospital. A standardized questionnaire was used to collect data during the interview when they visited their neurologist at the center. We recorded the demographic data such as name, age, and gender. The recorded clinical information consisted of the diagnosis, 24-h video electroencephalogram (EEG), neuroimaging, epilepsy type, seizure frequency, and medication profile. The establishment of the databank was approved by the Human Research Ethics Committees of our hospital, and participants provided written informed consent before we collected the data.

We retrospectively reviewed the medical records of patients in the databank from January 2020 to July 2021. Individuals were included in our analysis if they had the following criteria: (1) had a definite diagnosis of epilepsy in accordance with the International League Against Epilepsy (ILAE) (14); (2) had reached the age of 18 years or older; and lastly (3) had been taking a stable dose of an ASM for at least 1 month. We excluded individuals who had non-epileptic seizures, a severe neurological disorder other than epilepsy (e.g., Parkinson's disease), or a serious physical condition (e.g., a significant hepatic, renal, or cardiopulmonary condition). The records were examined by two investigators to ensure that individual data were not repeatedly entered in the analysis.

### Data Collection

To evaluate the history of SC, we defined SC as three or more seizures occurring within 24 h, which is the habitual pattern that has been widely used by previous studies (5, 6, 15). Data on potential risk factors for SC (i.e., age, sex, age at first seizure onset, duration of epilepsy, family history of epilepsy, history of febrile seizures, seizure frequency over the last 6 months, epilepsy type, multiple seizure types, number of anti-seizure medications (ASM), known etiology of epilepsy, abnormal MRI, drug-resistant epilepsy, and presence of status epilepticus) were collected and recorded from a databank. A family history of epilepsy was defined as epilepsy occurring in a first-degree relative (parent or sibling). Patients had a known etiology of epilepsy if they reported genetic, tumor, vascular, dysplasia, infectious, autoimmune, hippocampal sclerosis, or other defined etiologies. MRI findings were reported by a neuroradiologist, and their reports were subsequently reviewed by a second epileptologist, who made a final classification for this study. MRI was classified as abnormal only when the observed abnormality was considered the cause of epilepsy. Drug-resistant epilepsy was defined as a failure of adequate trials of two tolerated, appropriately chosen, and used ASM schedules (whether as monotherapies or as combination) to achieve sustained seizure freedom (16). SE was defined if the diagnostic time exceeded 5 min of ongoing seizure activity for convulsive SE or 10 min for absence status or focal status with or without impaired consciousness (17).

### Statistical Analysis

Data are described as percentages for categorical variables and as medians [interquartile ranges (IQRs)] for continuous variables. For group comparisons, Mann–Whitney *U*-tests were used for continuous variables, while Chi-square/Fisher's exact tests were used for categorical variables. We employed logistic regression models to investigate the relationships between the individual patient demographic/clinical variables and the history of SC. Variables achieving significance at *p* < 0.05 in the univariate analyses were entered into multivariate logistic regression models. The results were presented as odds ratios (ORs) with the corresponding 95% CIs. The type of epilepsy was classified as focal epilepsy or non-focal epilepsy, including unknown classification. The same logistic regression analyses were also performed based on epilepsy type. All *p*-values were two-sided, and statistical significance was set at *p* < 0.05. All data were analyzed in SPSS 26.0 (SPSS Inc., Chicago, IL, USA).

## Results

In total, 606 consecutive PWE were included in the final analysis, with a median age of 35 years and a male percentage of 53.5%. The demographic and clinical characteristics are shown in Table 1. The median age at first seizure onset and epilepsy duration was 25 and 4 years, respectively. A total of 25.9% (157/606) of the patients were seizure-free in 6 months. Focal epilepsy was the most common epilepsy type reported by 452 (74.6%) patients, and 5% of the patients had multiple seizure types. Overall, 268 (44.2%) of the patients experienced at least one seizure cluster.

**Table 1:** Demographic and clinical characteristics.

**Characteristics (*****n*** **= 606)**	
Age (Y), Median (IQR)	35 (24, 51)
Male gender, n (%)	324 (53.5)
Age at first seizure onset (Y), Median (IQR)	25 (16, 43)
Duration of epilepsy (Y), Median (IQR)	4 (2, 14)
Family history of epilepsy, n (%)	58 (9.6)
History of febrile seizures, n (%)	49 (8.1)
**Seizure frequency over the last six months, n (%)**	
Seizure-free	157 (25.9)
<1/month	225 (37.1)
≥1/month	224 (37.0)
**Epilepsy type, n (%)**	
Focal epilepsy	452 (74.6)
Generalized epilepsy	84 (13.9)
Unclassified epilepsy	70 (11.6)
Multiple seizure type, n (%)	30 (5.0)
Number of present ASM, Median (IQR)	1 (1, 2)
Known etiology of epilepsy, n (%)	235 (38.8)
Abnormal MRI, n (%)	246 (40.6)
Drug-resistant epilepsy, n (%)	182 (30.0)
Presence of status epilepticus, n (%)	168 (27.7)
Histroy of SC, n (%)	268 (44.2)

*Y, years; IQR, inter-quartile ranges; ASM, anti-seizure medications; SC, seizure clusters*.

Patients with a history of SC were more likely to be older (*p* = 0.021), have a longer epilepsy duration (*P* < 0.001), reported a family history of epilepsy (*p* = 0.041), and experienced a higher seizure frequency over the last six months (*P* < 0.001) than those without a history of SC. Multiple seizure types were more frequent in patients with a history of SC (*p* < 0.001), and their number of present ASMs were higher (*p* < 0.001). Patients who experienced SC were more likely to have a known etiology of epilepsy (*p* = 0.002), abnormal MRI (*p* = 0.011), and drug-resistant epilepsy (*P* < 0.001). There was an increased risk of the presence of status epilepticus in the SC group (*P* < 0.001) for details (see Table 2).

**Table 2:** Comparison of the subgroups with and without history of SC.

**Characteristics**	**Subgroup** **with history** **of SC** **(***n*** = 268)**	**Subgroup** **without history** **of SC** **(***n*** = 338)**	***P*** **-value**
Age (Y), Median (IQR)	37 (25, 53)	34 (23, 50)	0.021
Male gender, n (%)	140 (52.2)	184 (54.4)	0.59
Age at first seizure onset (Y), Median (IQR)	25 (15, 44)	24 (16, 42)	0.762
Duration of epilepsy (Y), Median (IQR)	6 (2, 18)	4 (1, 10)	<0.001
Family history of epilepsy, n (%)	33 (12.3)	25 (7.4)	0.041
History of febrile seizures, n (%)	24 (9.0)	25 (7.4)	0.485
**Seizure frequency over the last six months, n (%)**			
Seizure-free	39 (14.6)	118 (34.9)	<0.001
<1/month	69 (25.7)	156 (46.2)	
≥1/month	160 (59.7)	64 (18.9)	
**Epilepsy type, n (%)**			
Focal epilepsy	194 (72.4)	258 (76.3)	0.268
Generalized epilepsy	40 (11.8)	44 (16.4)	
Unclassified epilepsy	30 (11.2)	40 (11.8)	
Multiple seizure type, n (%)	25 (9.3)	5 (1.5)	<0.001
Number of present ASM, Median (IQR)	2 (1, 2)	1 (1, 2)	<0.001
Known etiology of epilepsy, n (%)	122 (45.5)	113 (33.3)	0.002
Abnormal MRI, n (%)	124 (46.3)	122 (36.1)	0.011
Drug-resistant epilepsy, n (%)	134 (50.0)	48 (14.2)	<0.001
Presence of status epilepticus, n (%)	104 (38.8)	64 (18.9)	<0.001

*Y, years; IQR, inter-quartile ranges; ASM, anti-seizure medications; SC, seizure clusters*.

Univariate logistic regression analyses were performed to investigate the associations between all demographic/clinical variables and a history of SC. The univariate regression models confirmed that these ten variables were associated with an increased OR for SC: age (OR: 1.012; 95% CI: 1.002–1.022; *p* = 0.021), duration of epilepsy (OR: 1.031; 95% CI: 1.015–1.048; *p* < 0.001), family history of epilepsy (OR: 1.758; 95% CI: 1.018–3.037; *p* = 0.041), seizure frequency (OR: 3.008; 95% CI: 2.37–3.818; *p* < 0.001), multiple seizure type (OR: 6.952; 95% CI: 2.586–18.153; *p* < 0.001), number of present ASMs (OR: 2.198; 95% CI: 1.74–2.777; *p* < 0.001), known etiology of epilepsy (OR: 1.664; 95% CI: 1.196–2.314; *p* = 0.002), abnormal MRI (OR: 1.525; 95% CI: 1.099–2.114; *p* = 0.011), drug-resistant epilepsy (OR: 6.042; 95% CI: 4.098–8.906; *p* < 0.001), and history of status epilepticus (OR: 2.715; 95% CI: 1.882–3.917; *p* < 0.001). The ten variables that were significant in the unadjusted univariate analysis were entered into the multivariate logistic regression analysis. In the multivariate logistic regression models, age (OR: 1.014; 95% CI: 1.002–1.027; *p* = 0.02), seizure frequency (OR: 2.08; 95% CI: 1.555–2.783; *p* < 0.001), multiple seizure type (OR: 5.111; 95% CI: 1.737–15.043; *p* = 0.003), number of present ASMs (OR: 1.533; 95% CI: 1.15–2.042; *p* = 0.004), drug-resistant epilepsy (OR: 1.987; 95% CI: 1.159–3.407; *p* = 0.013), and history of status epilepticus (OR: 1.903; 95% CI: 1.24–2.922; *p* = 0.003) were independent variables associated with a history of SC in PWE for details (see Table 3).

**Table 3:** Univariate and multivariate logistic regression analysis on factors associated with the occurrence of SC.

	**Unadjusted model**			**Adjusted model**		
	**OR**	**95% CI**	***P*** **-value**	**OR**	**95% CI**	***P*** **-value**
Age	1.012	1.002–1.022	0.021	1.014	1.002–1.027	0.02
Male gender	0.915	0.664–1.262	0.59	-	-	-
Age at first seizure onset	1	0.991–1.008	0.941	-	-	-
Duration of epilepsy	1.031	1.015–1.048	<0.001	1.011	0.992–1.03	0.266
Family history of epilepsy	1.758	1.018–3.037	0.041	1.807	0.951–3.435	0.071
History of febrile seizures	1.231	0.686–2.21	0.485	-	-	-
Seizure frequency over the last six months	3.008	2.37–3.818	<0.001	2.08	1.555–2.783	<0.001
Epilepsy type	0.932	0.737–1.178	0.553	-	-	-
Multiple seizure type	6.852	2.586–18.153	<0.001	5.111	1.737–15.043	0.003
Number of present ASM	2.198	1.74–2.777	<0.001	1.533	1.15–2.042	0.004
Known etiology of epilepsy	1.664	1.196–2.314	0.002	1.327	0.85–2.074	0.213
Abnormal MRI	1.525	1.099–2.114	0.011	1.023	0.659–1.589	0.918
Drug-resistant epilepsy	6.042	4.098–8.906	<0.001	1.987	1.159–3.407	0.013
History of status epilepticus	2.715	1.882–3.917	<0.001	1.903	1.24–2.922	0.003

*ASM, anti-seizure medications; SC, seizure clusters; OR, odds ratios; CI, confidence intervals*.

Similar multivariate logistic regression analyses were performed for patients with focal epilepsy and non-focal epilepsy. In the focal epilepsy group, four variables were found to be associated with a history of SC: seizure frequency (OR: 1.838; 95% CI: 1.317–2.564; *p* < 0.001), number of present ASMs (OR: 1.486; 95% CI: 1.082–2.043; *p* = 0.015), drug-resistant epilepsy (OR: 1.986; 95% CI: 1.094–3.607; *p* = 0.024), and a history of status epilepticus (OR: 2.194; 95% CI: 1.336–3.602; *p* = 0.002). In the non-focal epilepsy group, the only variable associated with an increased OR for SC was seizure frequency (OR: 3.088; 95% CI: 1.679–5.678; *p* < 0.001) for details (see Table 4).

**Table 4:** Multivariate-adjusted odds ratios for SC stratified by epilepsy type (focal epilepsy vs. non-focal epilepsy).

	**SC of focal epilepsy group**			**SC of non-focal epilepsy group**		
	**Adjusted OR**	**95% CI**	***P***	**Adjusted OR**	**95% CI**	***P***
Age	1.013	0.999–1.026	0.069	1.022	0.993–1.052	0.138
Duration of epilepsy	1.019	0.998–1.041	0.083	0.981	0.938–1.026	0.409
Family history of epilepsy	1.581	0.737–3.393	0.24	2.851	0.792–10.265	0.109
Seizure frequency	1.838	1.317–2.564	<0.001	3.088	1.679–5.678	<0.001
Number of present ASM	1.486	1.082–2.043	0.015	1.499	0.715–3.142	0.283
Known etiology of epilepsy	1.311	0.791–2.174	0.293	1.545	0.591–4.036	0.0.375
Abnormal MRI	1.165	0.708–1.918	0.547	0.545	0.2–1.486	0.235
Drug-resistant epilepsy	1.986	1.094–3.607	0.024	4.17	0.995–17.47	0.051
History of status epilepticus	2.194	1.336–3.602	0.002	1.115	0.443–2.809	0.817

*ASM, anti-seizure medications; SC, seizure clusters; OR, odds ratios; CI, confidence intervals*.

## Discussion

The present study analyzed the factors associated with the occurrence of SC in a sample of adults with epilepsy in northeastern China. There are three main findings of our study. First, SCs are common occurrences at our study center and were reported by 44.2% of this population of adults with epilepsy. Second, the multivariate analysis identified six risk factors associated with the occurrence of SC in our cohort. Patients with older age, higher seizure frequency, multiple seizure types, more current ASMs, drug-resistant epilepsy, and history of status epilepticus had greater likelihood of having SC. Third, risk factors for the occurrence of SC varied between the focal and non-focal epilepsy groups.

The reported prevalence of SC ranged from 20 to 83% in prior studies (6, 8, 9, 12). Compared with the prevalence rates reported by prior studies, the 44.2% of PWE with a history of SC in this study was higher than the 29.0% reported by Haut et al. (6) and the 14.9% reported by Chen et al. (5), but lower than the 83% reported by Ferastraoaru et al. (8) and the 61.5% reported by Haut et al. (18). The varying reported occurrence rates of SC may be partly explained by the imprecise clinical definition of SC across the studies. Another possible explanation could be the different study populations and the inclusion of PWE with varying degrees of severity of epilepsy across the studies. Additionally, there may be an overestimation of SC prevalence since our data were collected from a tertiary epilepsy center that has more patients with refractory epilepsy.

Significant risk factors for the occurrence of SC reported previously included female gender, drug-resistant epilepsy, head trauma, symptomatic generalized epilepsy, intractable epilepsy (defined as a lack of seizure control/higher seizure frequency/absence of one-year seizure freedom), duration of epilepsy, seizure frequency, localization (extratemporal epilepsy, in particular, frontal lobe epilepsy), MTS, remote symptomatic epilepsy, posttraumatic epilepsy, CNS infection, cortical dysplasia, and status epilepticus (4–6, 15, 18–25). Our findings confirmed the independent relationships between a history of SC and several risk factors, including seizure frequency, drug-resistant epilepsy, and history of status epilepticus. Our novel findings were that older age, multiple seizure types, and more current ASMs were significantly associated with seizure cluster occurrence in this cohort. Chen et al. also reported a link between 2 or more ASMs that did not work in the history of the patient's treatment for epilepsy and seizure cluster occurrence (5). Yao et al. predicted that multiple seizure types were associated with a decreased likelihood of seizure remission in PWE by using supervised machine learning technologies (26). In a recent case-control study of ASM-resistant patients and ASM-responsive controls, Brad et al. found that certain seizure-type combinations, such as a combination of absence, myoclonic, and generalized tonic-clonic seizures, were independent predictors of ASM resistance in PWE (27). The association between ASM resistance and seizure cluster occurrence has been established (10). Furthermore, univariate analyses found associations of a known etiology of epilepsy and abnormal MRI with the risk of SC. However, these associations disappeared in the multivariate analyses. Thus, a higher seizure frequency, more current ASMs, drug-resistant epilepsy, history of status epilepticus, and multiple seizure type—all features of severe epilepsy—appeared to be significantly associated with an increased likelihood of the occurrence of SC. In consideration of these findings, we proposed a hypothesis that the occurrence of SC in persons with epilepsy, to some extent, is determined by epilepsy severity.

To date, it remains unclear why seizures cluster. The neurobiological underpinnings of SC were not addressed in our investigation. However, prior evidence indicated that this may be because endogenous hormone fluctuations can lead to periods of enhanced seizure susceptibility and other periods with greater resistance to seizure recurrences (28). Periods of enhanced seizure susceptibility might result in the occurrence of SC (7).

Prior researchers identified focal epilepsy as a risk factor for SC in PWE (5). However, we could not replicate the previously reported relationship between epilepsy type and SC. When examining SCs by epilepsy type, Chen and his colleagues reported the differences in the prevalence rates and the related risk factors in patients across the different epilepsy types (5). Our results also supported this finding, and the risk factors for the occurrence of SC varied between patients with focal and non-focal epilepsy. Higher seizure frequency was the only consistent risk factor associated with a higher likelihood of developing SC in patients with focal and non-focal epilepsy.

Several limitations should be acknowledged in the current study. First, this study is limited by its retrospective observational nature. Data were collected based on patient self-reports, and thus, there may be recall bias. Second, the data were restricted to PWE in a tertiary epilepsy center, which may also cause a strong sampling bias toward people with refractory epilepsy. Another inherent bias of this study is that patients who died from SC cannot be included. Third, some factors that might be associated with the occurrence of SC in epilepsy [e.g., psychiatric symptoms, sleep quality, or extratemporal epilepsy (6)] were not available and were not investigated in this sample. It is well-known that patients with temporal lobe epilepsy usually have fewer SCs than extratemporal, especially with frontal lobe epilepsy. Finally, we did not differentiate between patients who had a single SC and those who had multiple SCs in this study.

## Conclusion

Seizure clusters are common occurrences at our study center and were reported by 44.2% of this PWE population. The occurrence of SC in persons with epilepsy, to some extent, is determined by epilepsy severity. Factors associated with the occurrence of SC included older age, higher seizure frequency, multiple seizure types, more current ASMs, drug-resistant epilepsy, and history of status epilepticus.

## Data Availability Statement

The raw data supporting the conclusions of this article will be made available by the authors, without undue reservation.

## Ethics Statement

The studies involving human participants were reviewed and approved by Human Research Ethics Committees of the First Hospital of Jilin University. The patients/participants provided their written informed consent to participate in this study.

## Author Contributions

RZ and WL conceived of and designed the study. RZ, QC, and XZ were involved in data acquisition. RZ and QC analyzed the data and wrote the manuscript. All authors contributed to the article and approved the submitted version.

## Funding

This work was supported by a grant from the Programme of Jilin University First Hospital Clinical Cultivation Fund (LCPYJJ2017006).

## Conflict of Interest

The authors declare that the research was conducted in the absence of any commercial or financial relationships that could be construed as a potential conflict of interest.

## Publisher's Note

All claims expressed in this article are solely those of the authors and do not necessarily represent those of their affiliated organizations, or those of the publisher, the editors and the reviewers. Any product that may be evaluated in this article, or claim that may be made by its manufacturer, is not guaranteed or endorsed by the publisher.
